# Randomized community trial on nosocomial infection control educational module for nurses in public hospitals in Yemen: a study protocol

**DOI:** 10.1186/s12912-019-0333-3

**Published:** 2019-03-19

**Authors:** Gamil Alrubaiee, Anisah Baharom, Ibrahim Faisal, Kadir Shahar Hayati, Shaffe Mohd. Daud, Huda Omer Basaleem

**Affiliations:** 1 Department of Applied Medical Sciences, Faculty of Medical Sciences, Al-Razi University, Sana’a, Yemen; 2 Department of Community Health, Faculty of Medicine and Health Sciences, Universiti Putra Malaysia, Seri Kembangan, Malaysia; 3 Department of Foundations of Education, Faculty of Educational Studies, Universiti Putra Malaysia, Seri Kembangan, Malaysia; 4 Department of Community Medicine and Public Health, Faculty of Medicine and Health Sciences, University of Aden, Aden, Yemen

**Keywords:** Nosocomial infections, Educational module, Training course, Public hospitals, Nurses in Yemen

## Abstract

**Background:**

Nosocomial infections remain a global health problem and they are considered as one of the leading causes of increased morbidity and mortality. In-service training courses related to infection control measures can help nurses to make informed and therapeutic decisions which could prevent or reduce the incidence of nosocomial infections. This study protocol is of a hospital-based trial to develop, implement and evaluate an educational module on nosocomial infection control among nurses in public hospitals in Yemen. This study is currently ongoing and at the analysis stage.

**Methods:**

A three-arm single-blinded randomized community hospital-based trial was conducted to evaluate the effectiveness of a newly developed nosocomial infection control educational module among nurses in public hospitals in Yemen. To ensure effective delivery and acquisition of knowledge, the Situated Learning Theory was applied during the course of the intervention. A total of 540 Yemeni in-ward nurses, who had three years nursing diploma and at least a year of working experience in the selected public hospitals were recruited in this study. The hospitals were the unit of randomization whereby eight hospitals were assigned randomly to intervention and waitlist groups. Intervention group-1 (*n* = 180) received an educational module supported by audio-video CD and a training course for eight weeks. Intervention group-2 (n = 180) was given only the educational module with audio-video CD (without the training course). The waitlist group received no intervention during the period of data collection but they will be given the same training and learning materials after the completion of the study.

**Discussion:**

This study contributes to the lack of a nosocomial infection control educational module for nurses in Yemen. It is hoped that the educational module will serve as an effective approach to increase the nurses’ knowledge and improve their practices regarding nosocomial infection control measures and hence decrease the prevalence of nosocomial infections in the future.

**Trial registration:**

ID: ISRCTN19992640, Date of registration: 20/6/2017. This study protocol was retrospectively registered.

**Electronic supplementary material:**

The online version of this article (10.1186/s12912-019-0333-3) contains supplementary material, which is available to authorized users.

## Background

Infections acquired by patients after 48 to 72 h during the process of receiving medical care in the hospital which was not present or incubating at the time of admission are defined as nosocomial infections (NIs). Such infections can also appear within 30 days of post-operative procedure, or within 90 days if there is an implant in place [1]. NIs have serious consequences and impact on the patient as they lead to prolonged hospitalization, additional medical care cost, psychological effects, work dismissal, and in the worst case, increased risk of morbidity and, mortality [2]. The consequences do not only affect the patients but also their families and the community as a whole. The family members might be affected financially if the patient is the breadwinner of the family, or the infection might spread to the family members as a result of close contact with the patient during visiting hours in the hospital. The community could be affected as a result of usage of public funds for additional medical costs needed as a consequence of prolonged hospital stay and management of NIs, which are initially potentially preventable conditions [2, 3].

Many previous studies have documented that poor nurses’ knowledge and malpractices regarding basic infection control measures were among the risk factors which increase the susceptibility of patients to be infected whilst receiving healthcare [4, 5]. Hence, education on nosocomial infection control and prevention measures among healthcare workers is important to reduce the incidence of NIs. Nurses, being one of the frontline healthcare workers, form the largest group of healthcare professions. They play an important role in ensuring the control and prevention of NIs. Previous studies have shown the important role of education and training courses in improving nurses’ knowledge and practices and consequently reducing the incidence of NIs. For instance, Nguyen, Nguyen [5] reported that the incidence of NIs reduced from 13.1 to 2.1% (84%) after conducting a hand hygiene educational program.

### Governmental goals in Yemen and contribution of this study

The Yemeni government, represented by the Ministry of Public Health and Population, has directed all public hospitals to be committed to the preparations for accreditation. The aim of the accreditation is to improve the capacity of national hospitals to enable them to provide quality care. One of the criteria to achieve the accreditation is the presences of an infection control program with qualified staff. Having trained nurses in the control and prevention of nosocomial infections will assist in the infection control program. Therefore, this study is in-line with the directive of the Ministry of Public Health and Population in which it will assess the Yemeni nurses’ knowledge and practices with regards to infection control measures and explore the aspects that need to be improved by the government. NIs can be prevented by implementing in-service training courses in order to improve nurses’ knowledge with regards to infection control measures and by enhancing their ability to apply this knowledge in the real situation. The present study involves the development of a nosocomial infection prevention and control training module and evaluation of the effectiveness of the module among nurses in public hospitals in Yemen.

### Study aim

This study aims to improve the nurses’ knowledge and practices related to prevention and control of NIs and subsequently reduce incidences of NIs in public hospitals in Yemen.

### Study objectives

- To determine the current level of nurses’ knowledge and practices related to NIs control measures at baseline.
- To determine the association between nurses’ knowledge scores and previous in-service training courses and previous working experience at baseline.
- To determine the association between nurses’ practices scores and previous in-service training courses and previous working experience at baseline.
- To develop an education module on NIs control measures for nurses.
- To implement the educational module among in-ward-nurses in public hospitals.
- To evaluate the effectiveness of NIs control educational module in improving nurses’ knowledge and practice at immediately after and 3 months post-intervention within and between intervention 1 and 2 and the waitlist groups, after controlling for covariates.

## Methods

### Study settings and population

This protocol reports an ongoing study which was conducted in Azal Region in Yemen. This study is currently at the analysis stage. There are five cities in Azal Region, namely Sana’a, Amanat Al-Asemah, Dhamar, Amran, and Sa’ada. A total of three cities was adequate to fulfill the required calculated sample size for this study. Simple random sampling was used to choose three cities out of the five. There was a total of eight public hospitals in the three cities, all of which were selected for this study.

### Design and main hypothesis

**Fig. 1:**
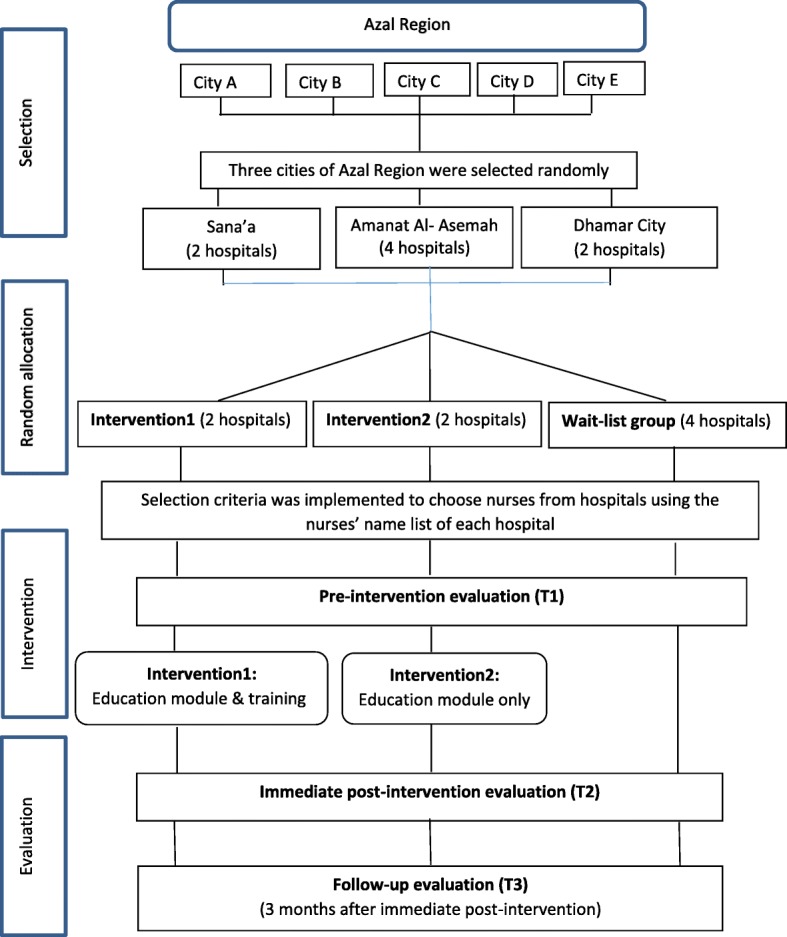
Flow chart of the study design and outcome evaluation

This study was conducted in three phases: (1) educational module and questionnaire development, (2) intervention implementation and (3) intervention evaluation. A three-arm cluster randomized community trial was used to evaluate the effectiveness of an educational module based intervention for prevention of nosocomial infections. Two of them are intervention groups, while the third one is a waitlist group (control group). Intervention group-1 received an educational module supported by audio-video CD and training course for 8 weeks. Intervention group-2 was given only the educational module with audio-video CD (without the training course), whereas the waitlist group received no intervention during the period of data collection. For ethical considerations, the wait list group was given the same training and learning materials after completion of the study. A self-administered questionnaire was also used to evaluate the knowledge and practice of the nurses at three points of times; baseline, immediately post-intervention and three months post-intervention. This study hypothesized that the intervention group-1 will score significantly higher than the intervention group-2 and the wait-list group on the immediate and three-month post-intervention evaluation of knowledge and practice scores of NIs control measures. The research framework of the study is presented in Fig. 1.

### Participants, setting, and procedure

The selection criteria for the participants were: a fully employed in-ward nurse in the selected public hospitals, originates from Yemen, has a 3-year diploma in nursing from a local institution, and has at least one year of working experience. Nurses from other countries of origin and Yemeni nurses who were trained overseas will receive the training after the module has been evaluated at the end of the study and when approved by the hospital authorities. The selected nurses were informed about the aims of the study and the intervention, methods of evaluation, and data protection using participants’ information sheet and consent. Prior to collecting the baseline data and conducting the intervention, an informed consent was taken from all participants and each participant was given a unique identification number to trace her participation at the different points of time during data collection.

### Randomization and blinding process

The eight selected hospitals were randomly coded as A, B, C, D, E, F, G, and H using the lottery method. These codes were masked by the investigator who then allocated the hospitals to one of the three waitlist/intervention groups. The names of the waitlist/intervention groups were sealed in opaque envelopes which were sequentially numbered to ensure allocation concealment and avoid any selection bias. Regarding this, a random number generator program (https://www.randomizer.org/) was used by an independent statistician to allocate the hospitals to one of the three waitlist/intervention groups. This is a single-blinded trial study as the hospitals and participants were blinded, i.e. they were not aware of the random allocation or the hypothesis that was tested until the intervention has started, after which blinding was not possible because the intervention hospitals had already received the training course. Likewise, group allocation was not released to the respondents prior to obtaining the written consent and collecting the baseline data.

### Sample size calculation

The sample size was estimated based on two sample group proportion [6].

$$ \mathbf{N}={\left[{\mathbf{Z}}_{\mathbf{1}-\boldsymbol{\upalpha} /2}\sqrt{\mathbf{2}\mathbf{P}\left(\mathbf{1}-\mathbf{P}\right)}+{\mathbf{Z}}_{\mathbf{1}-\boldsymbol{\upbeta}}\sqrt{\mathbf{P1}\left(\mathbf{1}-\mathbf{P}\mathbf{1}\right)+\mathbf{P}\mathbf{2}\left(\mathbf{1}-\mathbf{P}\mathbf{2}\right)}\right]}^{\mathbf{2}}/{\left(\mathbf{P}\mathbf{1}-\mathbf{P}\mathbf{2}\right)}^{\mathbf{2}}. $$

P1 = Proportion of knowledge among nurses in the intervention group 70% = 0.70; and P2 = Proportion of knowledge in the control group 50% =0.50 [7]. P = (P1 + P2)/2 = 0.6. The sample size in each arm was 93. As participating nurses were nested within hospitals, design effect was considered. Participants per hospital assumed 16 and an intra-cluster co-efficient (ICC) was 0.05. This resulted into a design effect = 1+ (m-1) × ICC = 1+ (16–1) × 0.05 = 1.75. By multiplying this result by the required sample size, *N* = 93, the sample size became, 93 × 1.75 = 163. Assuming attrition of 10%, *N* = 163+ 10% of 163 = 163+ 17 = 180. Thus, the total sample size in each arm, *N* = 180. Where, Z_(1-α/2)_, assumed 1.96, level of significance (α = 0.05; therefore 0.95 confidence level and Z_(1-β),_ 0.84, β = 0.20; 0.80 power was desired [8, 9].

### Intervention

The educational module was aimed to train the Yemeni nurses on the basic NIs control measures, who are then expected to apply the knowledge and skills to prevent the infection in the in-patient wards and provide safe nursing services. The content of the module was based largely on the Gulf Cooperation Council’s (GCC) infection prevention and control manual [10]. It focused on the basic infection control measures (Standard Precautions), which must be applied by the nurses to all patients in the in-patient wards all the time. At the end of this module, the nurses would be able to acquire the knowledge and skills needed to apply an appropriate nosocomial infection control measure during their daily practices in the hospitals. The module was divided into three units, namely introduction to nosocomial infection, prevention of person-to-person transmission, and prevention of transmission from the hospital environment. The sequence of the training was planned as such to ensure reinforcement of knowledge between the units’ topics, by reflection and group assessments. The teaching/learning strategies and assessment methods were consistent with the content and objectives of each topic in the module.

### Theoretical background

The method of delivery of the educational intervention was developed based on the “situated learning theory” which focuses on the learner as the center of the learning process. Previous studies using this theory have indicated that the situated learning theory, if integrated with a variety of teaching styles into educational programs, is capable of explaining the learning process as an integral part, connecting between the theoretical and practical aspects [4]. This theory comprises of four main aspects: content, context, a community of practice, and participation. Integration of these four aspects would enhance the problem-solving skills and critical thinking ability and eventually results in effective learning that lasts for a long time [11, 12]. All educational materials (printed training module, power point slides & training audio-video CD) were prepared and designed before the intervention period.

### Intervention elements

The educational intervention encompasses three units: with 20 h in total, spread over 1 month and a half and ten training sessions as follows:

- One hour for opening and pre-assessment.
- One hour for introducing the proposed educational module.
- One hour for an introduction to NIs.
- Nine hours of interactive lectures and audio-video demonstrations.
- Five hours of hands-on demonstrations of hand hygiene and the sequence of donning and removing PPE (2 + 3 h.).
- Two hours of situational-based scenarios.
- One hour for overall review and evaluation.

The learning process elements of situated learning theory, namely, content, context, community of practice and participation, were integrated through interactive learning between the participants and facilitators, and interchange of ideas and experiences among the participants. Based on this theory, the situation-based scenarios on topics related to infection control measures were prepared in the form of activities similar to the learners’ practices in real life situations. These sessions challenged their abilities to find solutions to these activities-related problems by sharing their experiences and working together in small groups. Consequently, learners’ problem-solving skills and critical thinking as well as the application of cooperative and participative teaching approaches for acquiring knowledge would improve.

Prior to implementing the intervention, a comprehensive communication strategy was put in place in order to ensure a successful implementation period. One infection control practitioner (ICP) in each hospital was appointed as a contact person. This person became responsible for regulating the planned activities as well as all contact between the hospital and the researcher. Moreover, the participants in the intervention were divided into 12 small groups, each of which included 15 participants. They were given two alternative choices to attain the training course during the week, either in the first 3 days or the second half of the week in order to accommodate different work patterns. By so doing, expected problems such as participants’ absence during training and refusal of administrators in the hospitals to conduct the training were overcome. The training course was delivered by three specialized nurses holding a master’s degree in nursing and has working experience in the field of infection control. A guest from the Central Sterile Supply Department (CSSD) also assisted in one specific training session. In this study, the trainers were aware of the intervention.

### Research instruments

A questionnaire was developed to evaluate the effectiveness of an educational module by assessing nurses’ knowledge and practices in regard to NIs control measures. Based on an extensive literature review of previous related researches, the developed questionnaire covers two dimensions: (1) prevention of person-to-person infection transmission (hand hygiene, use of PPE, safe injection practices, respiratory hygiene), and (2) prevention of transmission from the hospital environment (reprocessing of patient care equipment, routine hospital cleaning, safe linens handling, and safe hospital waste handling and disposal). Accordingly, NIs have many constructs and dimensions that must be taken into consideration in order to assess knowledge and practices. Hence, the questionnaire of this study was developed on the related theoretical background and a broad set of constructs and dimensions related to NIs control measures following an extensive literature search.

The questionnaire consists of three parts: (1) demographic data such as age, gender, previous in-service training course related to infection control measures, and previous working experience with patients having NIs, (2) a knowledge scale, with 30 items to elicit nurses’ knowledge about NIs control measures and (3) practice scale, consisting of 15 scenario-based items that focuses on the daily basic infection practices being implemented in the wards. “Correct”, “incorrect”, and “I don’t know” statements were used to evaluate nurses’ knowledge. Scenario-based questions with “Correct”, “incorrect”, and “I don’t know” responses were used to evaluate nurses’ practices. After correction of the reverse statements involved in the questionnaire, each “correct response” was allocated as 1; and “incorrect response” and “I don’t know” were allocated as 0.

Situated learning theory emphasizes that knowledge and skills are gained in the context of daily activities. The second and third part of the questionnaire integrated the theory by assessing the knowledge (second part of the questionnaire) and skills in their daily activities (third part of the questionnaire).

### Validity and reliability

The questionnaire on NIs control measures was developed in English language (Additional file 1). Translation to the Arabic language (Additional file 2) and back-to-back translation were performed to evaluate if the questionnaire still maintained its original ideas across the linguistic boundaries after translating it into another language, and after performing back translation into the original language [13]. The questionnaire was examined by six expert panellists to affirm its content validity. Selection of the experts was based on having at least 5 years’ experience working in the field of nosocomial infection prevention and control in either hospitals or academic institutions, and possessed at least a master’s degree in nursing or medical field. A Content Validity Index (CVI) form was distributed to the experts for this purpose. The experts’ comments were taken into consideration and changes were made to fit the experts’ recommendations. The results of the CVI evaluation revealed that the CVI for knowledge scale was 93%, and for the practice scale was 90%. This indicates that the scale has an acceptable level of validity. Exploratory factor analysis (EFA) was also used to assess the construct validity of the questionnaire. A pilot study was conducted in two public hospitals (not a part of the study hospitals) located in Al-Hodeida City, Yemen. Out of the 150 distributed questionnaires, 121 were subjected to an EFA which is a useful and widely accepted method to test construct validity with multiple dimensions [14].

Reliability of the questionnaire was measured using Cronbach’s coefficient alpha (α) test. The result showed that the knowledge scale was 0.81, and the practice scale was 0.79, which meant that both scales were acceptable. Furthermore, the questionnaire was pre-tested for comprehensibility of its items/ questions by distributing 20 copies among working nurses. The modifications and adjustments were made based on the responses in the pre-pilot study. The nurses who participated in the pre-test were excluded from the actual study sample.

### Data collection

Baseline data were evaluated 1 month prior to conducting the intervention using the developed questionnaire. The researcher and the coordinators in each hospital were responsible for distributing and collecting the data from the respondents. The same questionnaire was used to evaluate nurses’ knowledge and practices immediately after conducting the intervention, with re-arrangement of the sequence of the questions. This was to avoid the issue of those who had memorized the answers. A follow-up evaluation was conducted 3 months after the intervention. This evaluation was to ascertain whether there was any immediately realized gains in nurses’ knowledge and practices which sustained over the 3 months after the intervention. At the end of this phase, the respondents in the waitlist group received all the educational materials given to the intervention groups, as part of the ethical consideration.

### Study duration

The study was conducted over a period of 10 months. The activities of the educational intervention started from 1st May till 30 October 2016.

### Data analyses

Statistical Package for Social Sciences (IBMSPSS), version 22.0 was used for the purpose of data analysis in this study. The collected data was entered, cleaned and checked by assessing frequencies for all variables [15]. Exploration of data entering was done using SPSS to verify and ensure that there were no entering mistakes and detect missing data and outliers.

The proportion, mean and standard deviation were used to measure the level of knowledge and practice at baseline. Logistic regression was used to find out the association between the level of knowledge and practices of nurses and the selected variables at baseline. Statistical significance was reported at a *p*-value of less than 0.05 levels (two-tailed) with 95% confidence interval. T-test and Kruskal-Wallis were used to determine the differences between groups at baseline. Generalized estimating equation (GEE) was also employed as a robust popular choice in cluster data analysis to detect the differences between and within the study groups at three points of time [16].

## Discussion

This study is ongoing and it is currently at the analysis stage. The study aims to evaluate the effectiveness of the educational intervention. In contrast to many reviewed studies that have used either one group pre-test-post-test design or two groups to evaluate the effectiveness of the educational intervention, the current study used a three-armed randomized hospital-based trial design. Such design allows a fair comparison of several intervention groups because comparisons are made against the same control group and under a single protocol at one time. According to Jaki [17], a three-armed design can use the same relevant features such as inclusion/exclusion criteria. Multi-arm interventions have advantages over two-arm interventions because such design can assist in evaluating the effectiveness of new interventions faster and more efficiently [18], evaluate more intervention groups at once, reduce the administrative burdens and costs by using one multi-arm intervention rather than conducting several consecutive interventions, reduce the sample size required, and finally increase the power to detect which intervention is better than the control group [9]. Based on the above advantages, the purpose of using intervention-2 (module only) in the current study was to evaluate whether this intervention as a self-study module “without training” is effective in improving nurses’ knowledge and practice regarding NIs control measures. There is no possible risk of physical bodily harm to the participants because this study used only a questionnaire as a tool to collect data. However, the current study is limited to nurses and public hospitals. Private hospitals were not included because of several differing characteristics such as they tend to be more specialized in the type of services which they provide. In addition, private hospitals have a more established infection control and prevention program, and their nurses are regularly trained in infection control.

## Additional files

Additional file 1: Questionnaire 1 English Questionnaire (DOCX 68 kb)

Additional file 2: Questionnaire 2 Arabic Questionnaire (DOCX 848 kb)

## Abbreviations

CSSD: Central Sterile Supply Department
CVI: Content Validity Index
EFA: Exploratory factor analysis
GCC: Gulf Cooperation Council
GEE: Generalized estimating equation
IBM SPSS: International Business Machines Statistical Package for the Social Sciences
ICC: Intra cluster co-efficient
ICP: Infection prevention practitioner
NIs: Nosocomial infections
PPE: Personal protective equipment
