# Correction to: Randomized community trial on nosocomial infection control educational module for nurses in public hospitals in Yemen: a study protocol

**DOI:** 10.1186/s12912-019-0340-4

**Published:** 2019-04-18

**Authors:** Gamil Alrubaiee, Anisah Baharom, Ibrahim Faisal, Kadir Shahar Hayati, Shaffe Mohd. Daud, Huda Omer Basaleem

**Affiliations:** 1 Department of Applied Medical Sciences, Faculty of Medical Sciences, Al-Razi University, Sana’a, Yemen; 2 Department of Community Health, Faculty of Medicine and Health Sciences, Universiti Putra Malaysia, Seri Kembangan, Malaysia; 3 Department of Foundations of Education, Faculty of Educational Studies, Universiti Putra Malaysia, Seri Kembangan, Malaysia; 4 Department of Community Medicine and Public Health, Faculty of Medicine and Health Sciences, University of Aden, Aden, Yemen

**Correction to: BMC Nursing**

**https://doi.org/10.1186/s12912-019-0333-3**

In the original publication of this article [1], a formula’s format in the section of “Sample size calculation” was wrong and needs to be revised.

The correct formula should be:

$$ \mathbf{N}={\left[{\mathbf{Z}}_{\left(\mathbf{1}-\boldsymbol{\upalpha} \right)}\sqrt{\mathbf{2}\mathbf{P}\left(\mathbf{1}-\mathbf{P}\right)}+{\mathbf{Z}}_{\left(\mathbf{1}-\boldsymbol{\upbeta} \right)}\sqrt{\mathbf{P1}\left(\mathbf{1}-\mathbf{P}\mathbf{1}\right)+\mathbf{P}\mathbf{2}\left(\mathbf{1}-\mathbf{P}\mathbf{2}\right)}\right]}^{\mathbf{2}}/{\left(\mathbf{P}\mathbf{1}-\mathbf{P}\mathbf{2}\right)}^{\mathbf{2}}. $$
