# Supplementary material for: Randomized community trial on nosocomial infection control educational module for nurses in public hospitals in Yemen: a study protocol
Source: BMC Nurs. 2019 Mar 19;18:10. doi: 10.1186/s12912-019-0333-3 (PMC6425650; doi:10.1186/s12912-019-0333-3)
Supplement: Supplementary file 1 — Questionnaire 1 English Questionnaire (DOCX 68 kb) [file 12912_2019_333_MOESM1_ESM.docx]

**English questionnaire**

| **Ref.** |  |
| --- | --- |


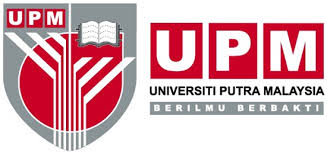


**QUESTIONNAIRE 1:**

**KNOWLEDGE AND PRACTICES OF NURSES REGARDING NOSOCOMIAL INFECTION CONTROL MEASURES IN**

**PUBLIC HOSPITALS IN AZAL REGION, YEMEN**

***SECTION I*: Personal data.**

**Please write down or tick (✓) in the box that corresponds to your response unless otherwise asked.**

| **A.** | **Socio-demographic characteristics.** |
| --- | --- |
| 1 | **Age**: …………. Years. |
| 2 | **Sex:** 1. 🞎 Male 2. 🞎 Female |
| 3 | **Marital status:** 1. 🞎 Currently married 2. 🞎 Currently unmarried |
| 4 | **Place of work**:………………………………………………………. |
| 5 | **Current position**: 1. 🞎 Clinical nurse 2.🞎 Dep. supervisor 3.🞎 Head of department  4.🞎 Others:……………………………………………. |
| **B**. | **In-service training courses in nosocomial infection.** |
| 1 | Have you ever participated in in-service education / workshops about “nosocomial infection control”?  1. 🞎 Yes 2.🞎 No |
| 2 | **If Yes,** When was the last course you attended?  1. 🞎 Less than one year 2.🞎 One year and more |
| **C.** | **Working experience.** |
| 1 | How long have you practiced as a nurse? …………….. Years. |
| 2 | Did you have experience with patients having nosocomial infection? 1. 🞎 Yes 2.🞎 No |
| 3 | **If Yes,** What kind of nosocomial infections did the patient had while you're caring/or clinical placement?  1.🞎 Respiratory tract infections. 2. 🞎 Blood-borne infections.  3.🞎 Surgical site infections. 4. 🞎 Gastrointestinal tract infections.  5.🞎 Urinary tract infections. 6. 🞎 Not sure.  7.🞎 Others ……………………………… |
| 4 | What’s your current specialty unit/department? ……………….. .. |

***SECTION II*: Knowledge about nosocomial infection control measures.**

**For each of the following questions, please tick (✓) in the box that corresponds to your answer.**

| **Statements** | | **Correct** | | **Incorrect** | | **Don’t know** | |
| --- | --- | --- | --- | --- | --- | --- | --- |
| 1 | Personal protective equipment is the most effective means of preventing and controlling nosocomial infections. | |  | |  | |  |
| 2 | Hand hygiene must be performed before and after any contact with the patient. | |  | |  | |  |
| 3 | Wash hands immediately before and after wearing gloves of any kind. | |  | |  | |  |
| 4 | Hand hygiene with antiseptic soap and running water should be done when hands are visibly soiled. | |  | |  | |  |
| 5 | When using alcohol-based hand rub, hand rubbing should continue for at least **20-30 Sec**. | |  | |  | |  |
| 6 | Gloves should be changed if they become contaminated or torn during contact with the same patient. | |  | |  | |  |
| 7 | The use of gloves can replace the need for hand hygiene. | |  | |  | |  |
| **Statements** | | **Correct** | | **Incorrect** | | **Don’t know** | |
| 8 | The mask must be changed if it becomes wet. | |  | |  | |  |
| 9 | The nurse can go from patient-to-patient wearing the same gown. | |  | |  | |  |
| 10 | The general sequence of putting on PPE is: Hand hygiene, gloves, gown, mask, eye protection. | |  | |  | |  |
| 11 | The peripheral **IV** cannula should be replaced after **72–96** hours as a maximum from its insertion. | |  | |  | |  |
| 12 | Change the needle when use the same mixing syringe to reconstitute several vials. | |  | |  | |  |
| 13 | A pre-soak cotton wools in a container with a **60–70**% alcohol is used to disinfect the skin at the injection site. | |  | |  | |  |
| 14 | Used needles should be bent immediately before disposed into a sharps waste container. | |  | |  | |  |
| 15 | Multi-patient use equipment (e.g. BP cuff, stethoscope, etc.) must be disinfected after each use by patients. | |  | |  | |  |
| 16 | Surgical instruments must be sterilized before re-use by another patient. | |  | |  | |  |
| 17 | Intermediate risk instruments require cleaning and sterilization or disinfection between uses by patients. | |  | |  | |  |
| 18 | Within normal circumstances, cleaning of low risk equipment is satisfactory. | |  | |  | |  |
| 19 | High risk zones in the hospital should be cleaned and disinfected daily. | |  | |  | |  |
| 20 | Patients' rooms and the bed's surroundings should be cleaned and disinfected prior to receiving a new patient. | |  | |  | |  |
| 21 | Telephones, door knobs, and surfaces like nurses counters need a daily disinfection. | |  | |  | |  |
| 22 | Alcohol solution is used for cleaning of blood spillages and other body fluids. | |  | |  | |  |
| 23 | Soiled linen should be collected and removed after each procedure on a daily basis or as needed. | |  | |  | |  |
| 24 | Soiled linen must be carefully rolled into the center and place in a cloth bag. | |  | |  | |  |
| 25 | Handle the used linen as infectious items even if there is no visible contamination. | |  | |  | |  |
| 26 | Segregation of the used linen at a point of use before sending to the laundry. | |  | |  | |  |
| 27 | The IV bags, tubing, and Foley's bags are considered from a high risk waste. | |  | |  | |  |
| 28 | Infectious hospital waste should be disposed in a black colored bag. | |  | |  | |  |
| 29 | Noninfectious hospital waste should be disposed in a yellow colored bag. | |  | |  | |  |
| 30 | Segregation of hospital waste (infectious &noninfectious) should not be done at a point of use. | |  | |  | |  |

***SECTION III*: Practices regarding nosocomial infection control measures.**

**For each of the following questions, please tick (✓) in the box that corresponds to your answer.**

Mrs. Fatima is a 60-year-old female. She was admitted to the surgical department on the morning of a planned cholecystectomy surgery. Mrs. Fatima has a history of obesity and diabetes. Post-operatively, it is planned that Mrs. Fatima will have a peripheral IV, a urinary catheter planned for 2 days. Her wound needs a dressing change by her nurse daily. In the department where Mrs. Fatima is staying, one patient on the unit is known to have vancomycin-resistant Enterococcus (VRE) and three other patients are known to have methicillin-resistant staphylococcus aureus (MRSA).

In this scenario, Mrs. Fatima as an elderly female patient and due to her obesity, diabetes, and the hospital environment and post-operative, she is at increased risk of acquiring nosocomial infections. As her primary nurse, your goal is to keep Mrs. Fatima free of nosocomial infection.

| **Statements** | | **Correct** | **Incorrect** | **Don’t know** |
| --- | --- | --- | --- | --- |
| 31 | I shall work under contact precautions all the time when I care Mrs Fatima. |  |  |  |
| 32 | If I had worn gloves to nurse Mrs. Fatima, I do not need to wash my hands immediately after glove removal. |  |  |  |
| 33 | I shall follow aseptic technique (HH, wearing sterile gloves and site disinfection) for insertion of peripheral venous catheter. |  |  |  |
| 34 | Conditions such as VRE and MRSA can't be transmitted through direct contact. |  |  |  |
| 35 | I shall follow aseptic technique (HH, wearing clean gloves and site disinfection) for urinary catheter insertion. |  |  |  |
| 36 | Be sure to clean the patient's skin first and then disinfected with alcohol before giving **IM** and **IV** injection. |  |  |  |
| 37 | I shall recap the needle before disposing of the syringe and the needle into the sharps waste container. |  |  |  |
| 38 | I shall clean the top of multi-use insulin vial with a 60–70% alcohol swab and hand hygiene before each use. |  |  |  |
| 39 | As possible, I shall expose the wound for a minimum time to avoid contamination. |  |  |  |
| 40 | I shall change the wound dressing at least 10 minutes after bed making and room cleaning procedures. |  |  |  |
| 41 | I shall allocate special tools to nurse Mrs. Fatima or clean and disinfect or sterilize these equipment after each use. |  |  |  |
| 42 | I shall disarm venous catheters if any sign of infection occurs, or if it appeared no longer needed for use. |  |  |  |
| 43 | I shall wear gloves and gowns all the time when in contact with Mrs. Fatima. |  |  |  |
| 44 | I shall not permit any sick-visitor to visit Mrs. Fatima. |  |  |  |
| 45 | I shall dispose the dressing material from Mrs. Fatima in a yellow plastic bag. |  |  |  |

Thank you for taking the time and effort to complete this questionnaire. Your assistance in providing information is very appreciated.

**The researcher**
