# Supplementary figures and images for: Randomized community trial on nosocomial infection control educational module for nurses in public hospitals in Yemen: a study protocol
Source: BMC Nurs. 2019 Mar 19;18:10. doi: 10.1186/s12912-019-0333-3 (PMC6425650; doi:10.1186/s12912-019-0333-3)

**Questionnaire 2**

**Arabic translation of the questionnaire**


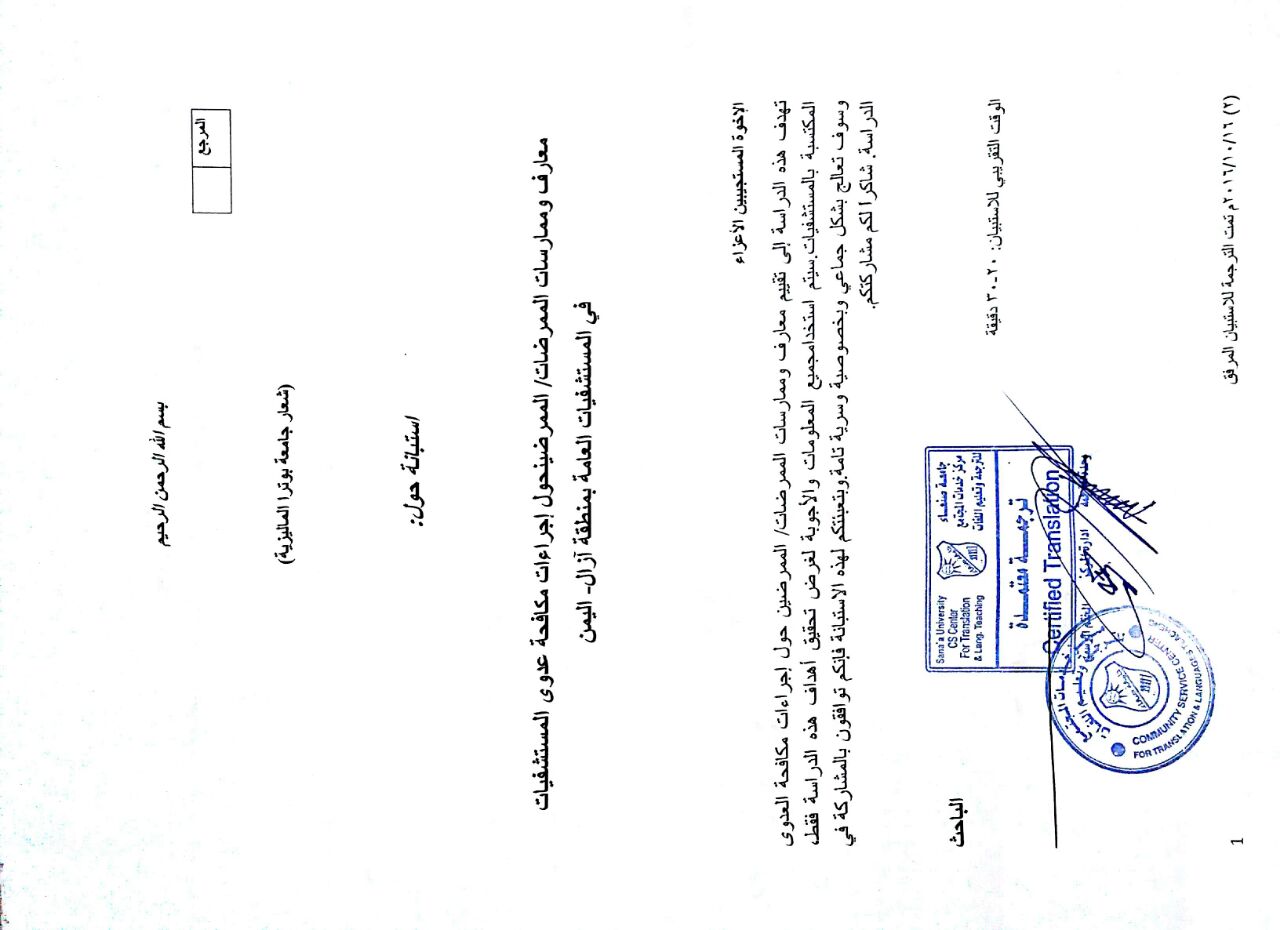


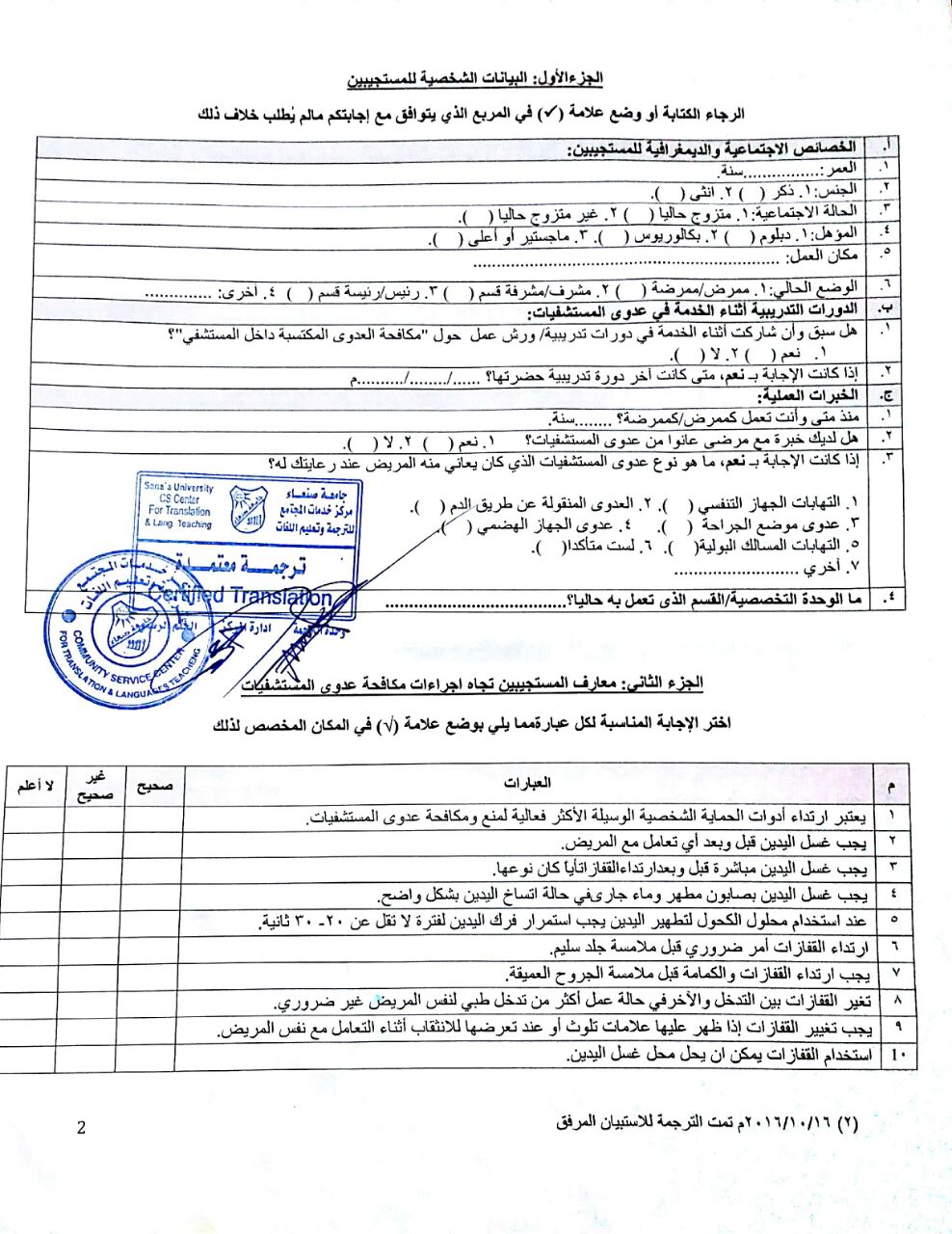


**
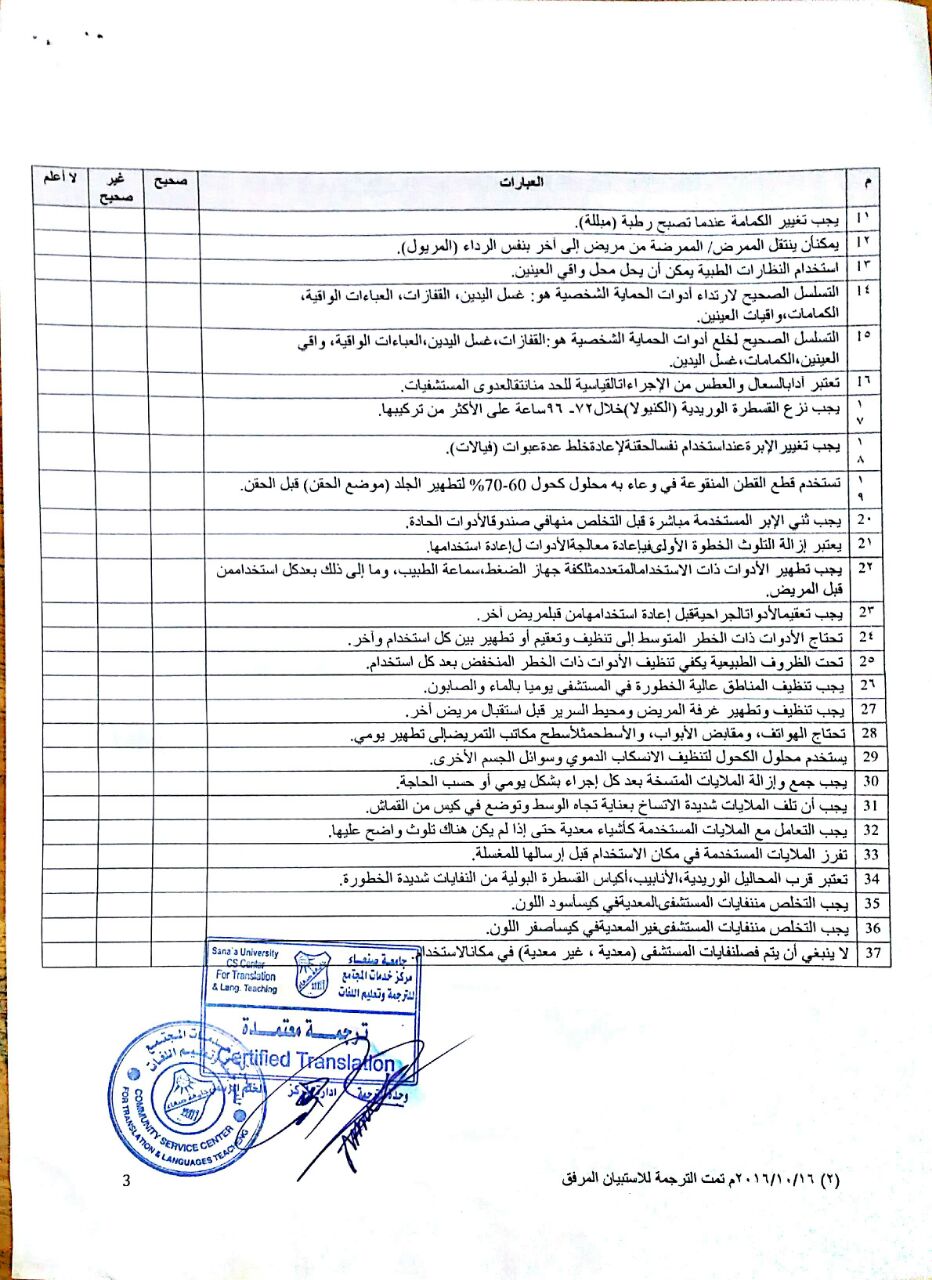
**

**
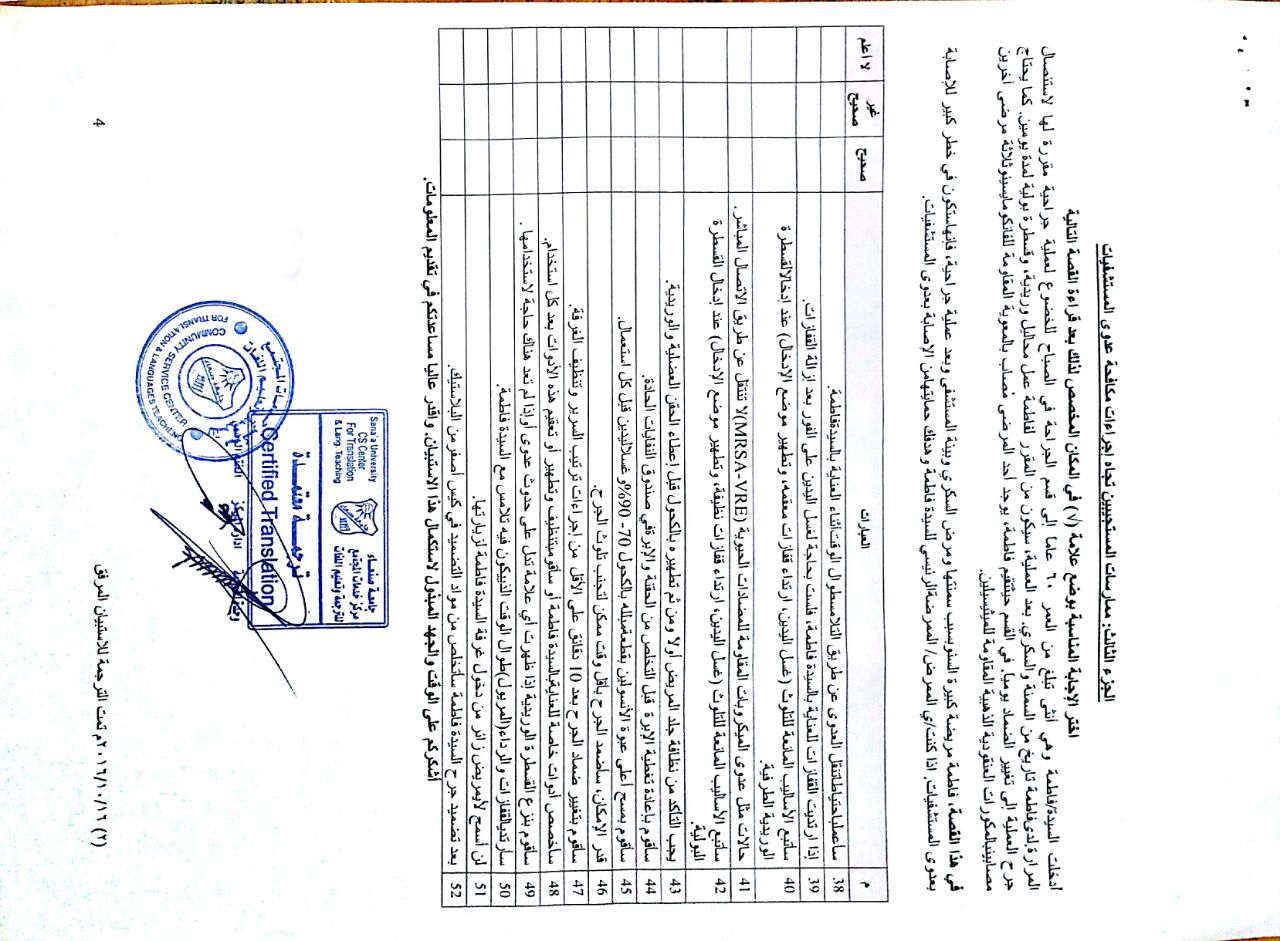
**

Supplement: Supplementary file 2 — Questionnaire 2 Arabic Questionnaire (DOCX 848 kb) [file 12912_2019_333_MOESM2_ESM.docx]
